# Symptoms and Comorbidities Differ Based on Race and Weight Status in Persons with HIV in the Northern United States: a Cross-Sectional Study

**DOI:** 10.1007/s40615-022-01271-0

**Published:** 2022-03-10

**Authors:** Kierra R. Butler, Faye R. Harrell, Bridgett Rahim-Williams, Jeffrey M. Robinson, Xuemin Zhang, Adwoa Gyamfi, Judith A. Erlen, Wendy A. Henderson

**Affiliations:** 1 Indiana University School of Medicine, Indianapolis, IN USA; 2 The Catholic University of America, Washington, DC USA; 3 Research Administration, Office of Research and Sponsored Programs, University of North Florida, Jacksonville, FL USA; 4 University of Maryland Baltimore County, Baltimore, MD USA; 5 Office of the Director, National Institutes of Health, Bethesda, MD USA; 6 School of Nursing, University of Connecticut, Storrs, CT USA; 7 School of Nursing, University of Pittsburgh, Pittsburgh, PA USA; 8 School of Medicine, University of Connecticut, Farmington, CT USA

**Keywords:** HIV, HAART, Cardiovascular, Hypertension, Gastrointestinal symptoms, BMI

## Abstract

**Background:**

Persons with HIV (PWHIV) on highly active antiretroviral treatments (HAART) may require specialized care based on health and demographic indicators. This study investigated the association of comorbidities, race, weight status, and gastrointestinal (GI) and cardiovascular (CV) symptoms among PWHIV.

**Methods:**

The Symptom Checklist, Co-Morbidity Questionnaire, and Sociodemographic Questionnaire were used to assess weight status and GI and CV symptoms among 283 PWHIV. Data were analyzed using latent class analysis on John’s Macintosh Project 13 Platform.

**Results:**

Participants were majority Black (50%), 69% male, and 35% AIDS diagnosed. Ages were 25 to 66. Clusters included least symptomatic status, weight gain, and weight loss by Black and non-Black participants. The non-Black weight gain cluster reported a higher incidence of AIDS (70.6% vs 38.2%), nausea (70.6% vs 17.6%), diarrhea (70.6% vs 26.5%), and shortness of breath (58.8% vs 20.6%) compared to the Black weight gain cluster. The Black weight loss cluster reported a higher incidence of CV symptoms such as chest palpitations (42.2% vs 2.7%), chest pain (44.4% vs 8.1%), and shortness of breath (73.3% vs 35.1%). Moreover, the Black weight loss cluster reported a higher incidence of all GI symptoms with the most prominent being diarrhea (71.1% vs 48.6%) compared to the non-Black weight loss cluster.

**Conclusions:**

The existing racial disparities in health-related quality of life for PWHIV may be improved through precision health and nutrition modifications. Continued research is needed investigating differential health outcomes among PWHIV on HAART.

**Clinical Trial Registration Number:**

NCT00222716. Registered 22 September 2005. Retrospectively registered, https://clinicaltrials.gov/ct2/show/NCT00222716?term=NCT00222716&draw=2&rank=1

## Background

Human immunodeficiency virus (HIV), a viral infection previously known as the wasting disease, has become a chronic yet manageable disease due to the advent of highly active antiretroviral treatments (HAART) [1]. HIV is characterized by chronic systemic inflammation including inflammation of the gastrointestinal (GI) barrier [2]. The virus depletes specific cluster of differentiation 4 [CD4+]  thymus cells [T-cells] in the gut-associated lymphoid tissue causing the intestinal barrier to become inflamed, leaky, and more permeable [3]. Inflammation in the gut may contribute to persons with HIV (PWHIV) having GI symptoms, such as nausea, vomiting, and diarrhea. Many of these symptoms cause weight changes, affecting adherence to HAART [4]. As the gut becomes more permeable, microbes can translocate into the systemic circulation and cause systemic inflammation. Chronic systemic inflammation may contribute to the progression of the disease, systemic dysregulation, and cardiovascular (CV) instability [5]. Microbial translocation is associated with hypertension and GI symptoms in PWHIV [6]. Hypertension and the inflammatory process driven by HIV alter the endothelial function of these patients [5].

Although HAART has prolonged life, studies suggest that it may also be associated with weight gain [7–9], CV disease, and GI symptoms [4, 7]. Weight gain after initiating HAART occurs frequently and is associated with lower mortality. Thus, it is looked upon as a favorable outcome [9]. However, excessive weight gain may lead to an increased risk of chronic conditions such as hypertension, diabetes mellitus, and CV disease [7]. In fact, CV disease is the leading cause of death among PWHIV [8].

Current literature has shown that weight changes in PWHIV on HAART have significant implications for health outcomes. CV disease and PLWHIV on HAART must have ongoing investigations to identify factors that will guide care over the lifespan. Gastrointestinal symptoms such as nausea, vomiting, diarrhea, and loss of appetite affect health outcomes for PWHIV on HAART. Increases in body mass index (BMI) are common in HIV-positive minorities and women. Symptoms were found to vary with patient race, age, and disease progression [9, 10]. Racial differences in conjunction with symptom presentation influence treatment options. Socioeconomic status and neighborhood disadvantage contribute to chronic stress for many Black/African American PWHIV and must be considered in their care when relevant [11, 12]. The clinician selects the proper treatment based on weight status (e.g., weight gain, weight loss), race, and symptoms (especially loss of appetite, acquired immunodeficiency syndrome [AIDS] classification, diarrhea, vomiting, and overeating). The selection of a specified treatment option is of paramount importance because the grid of care options varies; care options even oppose one another for certain groups. The care for Black/African American PWHIV on HAART who lose weight and have a loss of appetite is quite different than the care for Black/African American PWHIV on HAART who gain weight and overeat. Thus, precision care involving the proper choice of investigation and treatment improves patients’ outcomes and accuracy of care provided by practitioners [13].

Limited clinical research exists on the topics of changes in weight status, race classification, gastrointestinal health, and CV health among PWHIV on HAART. Clinicians benefit from knowledge regarding this population that will guide personalized care that targets each specific demographic group based on the needs associated with their weight status, their demographics, and presenting symptoms. The health disparities for Black/African American patients in terms of CV disease and HIV are well documented. There is a higher burden of CV risk factors and CV disease in patients who are Black/Africa American [14]. Additionally, it is 13 times more likely that a Black/African American over the age of 50 will receive an HIV diagnosis than a White/Caucasian over 50. To address these disparities, health professionals must provide ongoing and personalized care for this population [15]. As such, monitoring of PWHIV on HAART may require stratification and personalization based on demographics and symptoms. The differences found may be attributable to patients’ race, sex, weight status, comorbidities, and presenting symptoms. Moreover, as PWHIV on HAART age, comorbidities require closer examination, especially in terms of racial differences [16].

Current literature associated with weight status and multiple morbidities in PWHIV either examines the change in weight status/body mass index (BMI) related to race after HAART initiation [17], changes in BMI across a life span [18], existence of multiple morbidities and obesity without examining the effects of race[19], or multiple morbidities and aging [20], with some attention to weight/obesity [8]. Although research has been conducted on this topic, there is limited clinical research investigating these variables in a clinical population. Most research has included epidemiological large, cross-sectional studies and literature reviews. The current study was a secondary data analysis of a clinical population. The aim of the study was to investigate the association of race (Black and non-Black [Asian, mixed-race, and Whites]), weight status (weight gain and weight loss), gastrointestinal and cardiovascular symptoms (nausea, vomiting, shortness of breath, chest pain), and comorbidities (hypertension, coronary artery disease, heart failure) among PWHIV/AIDS. The findings of this study will contribute to the growing body of research addressing adverse effects experienced by PWHIV on HAART and provide important information related to personalized treatment, especially related to race.

## Methods

A secondary analysis was performed on data from the parent study, Improving Adherence to Antiretroviral Therapy (R01 NR04749, PI, J. A. Erlen, University of Pittsburgh). The aim of the study was to improve adherence to antiretroviral therapy in PWHIV through a nurse-delivered, telephone-based intervention. The parent study recruited 356 PWHIV on HAART from the Northern United States, specifically, western Pennsylvania and eastern Ohio community hospitals, university-based clinics, comprehensive HIV care centers, and through self-referral [21]. Inclusion criteria for the parent study required a positive HIV diagnosis by a healthcare provider being treated with antiretroviral medication, access to a telephone, and consent to participate in the study.

In this cross-sectional, secondary analysis, participants were included who submitted responses to all CV and GI symptoms and comorbidity questions on the Symptom Checklist, Co-Morbidity Questionnaire, and Sociodemographic Questionnaire (Center for Research in Chronic Disorders, University of Pittsburgh School of Nursing, 1999). Applying these inclusionary criteria to the parent study sample resulted in 283 participants for the current study.

**Fig. 1:**
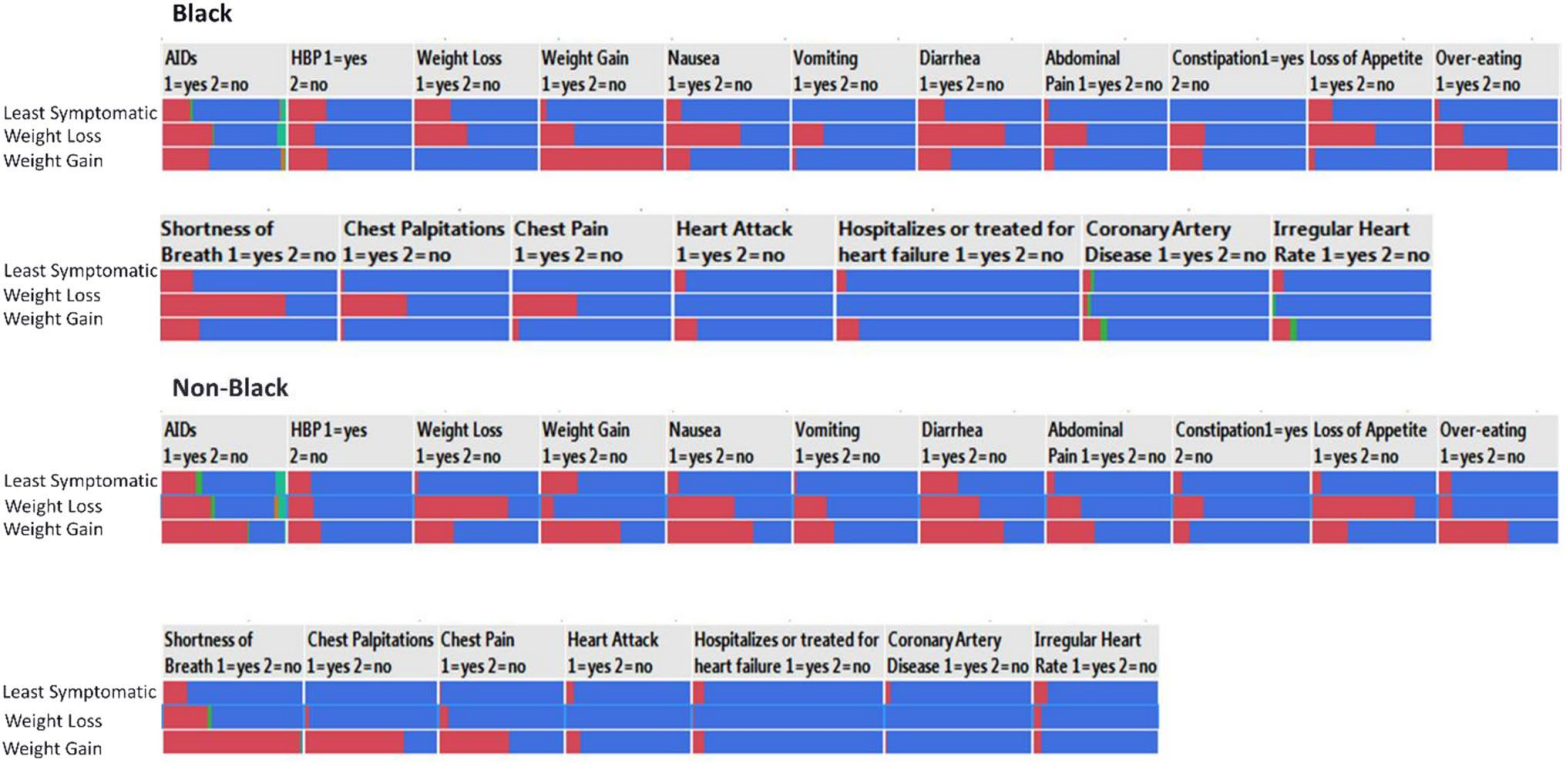
Latent class analysis results

To analyze data from the 283 participants, latent class analysis (LCA) was implemented with John’s Macintosh Project (JMP) 13 to perform an analysis of self-reported data on the three aforementioned questionnaires (Fig. 1). LCA is an unsupervised, multivariate grouping method that fits a model and determines the most likely “latent class” of each participant, in a pre-selected number of discreet classes (Statistical Analysis System [SAS] Institute Inc., 2016). In this analysis, three groups were selected a priori.

## Results

Of the participants, approximately 50% self-identified as Black, 69% as male, and 35% as having AIDS. Participants’ ages ranged from 25 to 66 years (mean age = 43.70 years) as shown in Table 1. Participants were grouped into clusters by race, Black and non-Black. Within the Black and non-Black groups, a pattern developed among the three clusters. Each racial group had a cluster of PWHIV who reported the lowest incidence of symptoms (weight loss, weight gain, vomiting, etc.) and comorbidities and a cluster characterized with a high incidence of weight gain and weight loss. Thus, each cluster was labeled based on the most prevalent symptom. Within each racial group (Black and non-Black), the clusters were the least symptomatic cluster, weight gain cluster, and weight loss cluster. After the LCA was conducted, descriptive statistics were calculated using International Business Machines Corporation Statistical Package for the Social Sciences (IBM SPSS) version 25 to ascertain the overall and average number of self-reported symptoms and comorbidities for each cluster within the Black and non-Black groups. A chart of the percentage of all GI and CV symptoms and comorbidities reported can be found in Table 2.

**Table 1:** Demographic characteristics of sample

*Demographic measures*		*Overall group* (*N* = 283)	*Black* (*n* = 144)	*Non-Black* (*n* = 139)
*Gender % (N)*	Male	68.90 (195)	32.51 (92)	36.40 (103)
	Female	31.10 (88)	18.37 (52)	12.72 (36)
*AIDS % (N)*	Male	34.98 (99)	11.31 (32)	13.78 (39)
	Female		5.30 (15)	4.59 (13)
	Age	Mean = 43.69		
	CD4 + count	Mean = 436.03		

CD4 count *N* = 182

**Table 2:** Cluster demographic and variable percentages

	Black least symptomatic cluster n = 65 (%)	Non-Black least symptomatic cluster n = 85 (%)	Black weight loss cluster n = 43 (%)	Non-Black weight loss cluster n = 37 (%)	Black weight gain cluster n = 34 (%)	Non-Black weight gain cluster n = 17 (%)
Sex	70.8 male	76.5 male	66.7 male	70.3 male	47.1 male	70.6 male
Age ± Std Dv	44.49 ± 8.8	45.54 ± 8.81	41.07 ± 7.3	44.24 ± 7.76	41.32 ± 5.50	41.94 ± 6.82
Mean CD4 ± Std Dv	496.34 ± 313.09	420.92 ± 231.72	345.16 ± 302.99	425.73 ± 308.46	494.31 ± 311.10	500.73 ± 269.75
AIDS	23	29.4	42.2	40.5	38.2	70.6
Mean BMI	26. 15 ± 6.10	25.4 ± 4.66	25.40 ± 6.75	23.86 ± 5.30	29.82 ± 5.24	27.94 ± 5.82
High blood pressure	32	17.6	20	21.6	32.4	29.4
Weight loss	32.3	2.4	42.2	83.8	0	29.4
Weight gain	1.5	30.6	28.9	10.8	100	64.7
Nausea	13.8	10.6	62.2	56.8	17.6	70.6
Vomiting	0	2.4	26.7	27	2.9	35.3
Diarrhea	23.1	31.8	71.1	48.6	26.5	70.6
Abdominal pain	4.6	7.1	35.6	27	8.8	41.2
Constipation	0	7.1	28.9	21.6	20.6	11.8
Loss of appetite	20	8.2	55.6	83.8	5.9	29.4
Overeating	4.6	10.6	24.4	10.8	55.9	58.8
Shortness of breath	20	17.6	73.3	35.1	20.6	100
Chest palpitations	1.5	0	42.2	2.7	0	82.4
Chest pain	0	1.2	44.4	8.1	2.9	58.8
Heart attack	7.7	5.9	0	0	14.7	11.8
Hospitalized or treated for heart failure	4.6	5.9	0	0	8.8	5.9
Coronary artery disease	4.6	3.5	2.2	0	8.8	0
Irregular heart rate	7.7	11.8	0	5.4	11.8	5.9
Heart valve disorder	1.5	4.7	4.4	0	5.9	0

### Least Symptomatic Clusters

Participants in both Black and non-Black least symptomatic clusters reported a lower incidence of GI and CV symptoms and comorbidities (heart attack, irregular heart rate) compared to the weight gain and weight loss clusters. However, participants in the Black least symptomatic cluster reported a higher incidence of high blood pressure (32.0% vs 17.6%) and weight loss (32.3% vs 2.4%) than their non-Black cluster counterparts. Weight gain (30.6% vs 1.5%), diarrhea (31.8% vs 23.1%), and AIDS (29.4% vs 23.1%) were reported more in the non-Black least symptomatic cluster compared to the Black least symptomatic cluster.

### Weight Loss Clusters

Participants who self-identified as Black in the weight loss cluster reported a higher incidence of all GI symptoms than non-Blacks with the most prominent being diarrhea (71.1% vs 48.6%) and nausea (62.2% vs 56.8%). CV symptoms including chest palpitations (42.2% vs 2.7%), chest pain (44.4% vs 8.1%), and shortness of breath (73.3% vs 35.1%) were more common in the Black cluster compared to the non-Black cluster. Interestingly, there were few to no reports of CV comorbidities (i.e., heart attack, heart failure, coronary artery disease) in the non-Black group. The most prominent symptoms of the non-Black weight loss cluster compared to the Black weight loss cluster were loss of appetite (83.8% vs 55.6%) and weight loss (83.8% vs 42.4%). Although participants in the non-Black cluster reported some CV symptoms (shortness of breath, chest palpitations, and chest pain), the incidence of CV comorbidities (irregular heart rate 2.5%) was very low.

### Weight Gain Clusters

Results revealed a high incidence of weight gain and overeating among participants in the weight gain clusters. Compared to the Black weight gain cluster, the non-Black weight gain cluster reported the highest incidence of AIDS (70.6% vs 38.2%), nausea (70.6% vs 17.6%), diarrhea (70.6% vs 26.5%), and shortness of breath (58.8% vs 20.6%). The Black weight gain cluster reported low incidence of GI symptoms (i.e., vomiting, abdominal pain), but a higher incidence of CV comorbidities than any other cluster (14.7%).

## Discussion

The aim of this study was to investigate the association among race (Black, non-Black), weight status, (weight gain and weight loss), and symptoms/comorbidities in PWHIV. With advances in medications used to treat and manage HIV, PWHIV are living longer [22]. However, longevity of life predisposes individuals to developing chronic disease conditions common in aging [23]. Additionally, long-term use of HAART by individuals living with HIV can affect weight [17]. This combination of multiple morbidities and weight status affects health-related quality of daily life for PWHIV.

### Multiple Morbidities and Microbial Translocation/Disease Progression

Antiretroviral treatments aid in replenishing the CD4+ T-cell count and decreasing the viral load, which reduces inflammation in the gut-associated lymphoid tissue and promotes immune reconstitution. Results from the administered surveys suggested that the Black and non-Black participants in the least symptomatic clusters were able to manage their HIV status. Of the participants in the Black and non-Black least symptomatic cluster, 23% vs 29.4%, respectively, reported an AIDS diagnosis. The lower incidence of AIDS had an association with fewer reports of GI and CV symptoms and comorbidity compared to the weight gain and weight loss clusters. This finding may indicate a reduced occurrence of the translocation of microbes and/or restored or improved CD4+ T-cell count due to an early initiation of HAART [24]. It may also be possible to surmise that the absence of an AIDS diagnosis is indicative of the efficacy and tolerability of HAART in participants in the least symptomatic cluster, which in turn suppresses the progression of HIV.

### Race/Ethnicity and Weight Status

In this study, PWHIV differed by race, weight status, and types of chronic disease conditions. Race included Black and non-Black participants. Weight status of the weight loss cluster and weight gain cluster differed by race in terms of GI and CV symptoms and comorbidities. While both Black and non-Black participants had increased weight loss and loss of appetite for the weight loss cluster, Black participants experienced the most incidence of diarrhea and non-Black participants had the least amount of CV symptoms. The weight gain cluster for Black participants had decreased GI symptoms with only one participant reporting vomiting. The non-Black weight gain group had a higher percentage of AIDS diagnosis. Several studies [17, 25] have likewise found these differences by race/ethnicity, comorbidities, and weight status among PWHIV.

The findings of this study related to the racial differences of symptoms within the same weight status cluster help to elucidate the need for precision healthcare. Precision care should also incorporate stress of neighborhood disadvantage and employment/socioeconomic status that often affects Black PWHIV. The practitioner examining a Black PWHIV that is having weight gain must consider CV symptoms and comorbidities, neighborhood disadvantage, and employment/socioeconomic status (SES) [11]. The non-Black PWHIV needs consideration for GI and CV symptoms and comorbidities along with an AIDS diagnosis. Simply treating PWHIV based on history may be ineffective as it may omit the required investigation and treatment required by each racial group [13].

Additionally, among the participants diagnosed with AIDS in this study, non-Black men had the highest prevalence of the disease. However, according to the Centers for Disease Control and Prevention, Black men have the highest prevalence of AIDS diagnoses [26]. This atypical burden of a higher number of reported AIDS diagnoses in non-Black males may be attributable to more non-Black males with AIDS enrolled in the study compared to their Black male counterparts (52% vs 47%). The higher prevalence of non-Black men with AIDS enrolled in this study could also be indicative of factors such as later diagnosis and treatment [27], or a lower tolerability of HAART.

### HAART and Weight

Initiating HAART has been associated with increasing BMI, and long-term use can lead to obesity [28]. The Black weight gain cluster had a higher BMI than the non-Black weight gain cluster (29.8% vs 27.9%). The higher BMI and self-reported weight gain could be attributed to a “return to health,” depression, or other factors such as unemployment [25] or metabolic syndrome [29]. According to several studies [17, 25], race attributed to the differences between weight gain and BMI. Participants who identified as Black were found more likely to have higher BMI and weight gain than their counterparts. The observed differences may be due to higher CD4+ T-cell count, a longer duration of HAART [7], and/or a higher pretreatment CD4+ T-cell count [17]. The chronic stress of neighborhood disadvantage and SES are associated with increased BMI and may contribute to the increased BMI for Black participants [11, 12]. The non-Black weight gain group reported a higher incidence of GI symptoms (loss of appetite, diarrhea, vomiting) compared to their counterparts in the Black weight gain group. Such symptoms typically are associated with weight loss [30]. A lower BMI has been shown to be associated with a higher mortality risk for patients on HAART [28].

Gastrointestinal symptoms such as nausea, vomiting, weight loss, and diarrhea are common in PWHIV [4, 31]. Although such symptoms were reported in both the weight loss and weight gain clusters, these symptoms were reported more frequently in the weight loss clusters. The GI symptoms could be attributable to the disease itself or a side effect of HAART medication [32]. A possible explanation for the higher incidence of weight loss and loss of appetite in the non-Black weight loss cluster could be a combination of multiple factors such as early vs late HAART initiation, antiretroviral efficacy and tolerability, disease progression, and race.

### Strengths

This study adds to the growing body of literature investigating health status among PWHIV. Our findings contribute new knowledge to the limited research examining the role of weight and its effects on GI symptoms and cardiovascular risks among PWHIV. Insights into the relationships among this tetrad (race, weight, GI symptoms, and cardiovascular risks) suggest a call for primary prevention to address and promote healthy weight status to improve health-related quality of life for PWHIV. The study reported herein purposefully implemented the latent class analysis (LCA), a type of structural equation modeling, to find groups or subsets of cases within the multivariant categorical data. Many analyses use race as a covariant and adjust for race and ethnicity and outcomes, thereby not purposefully including race and ethnicity as the main effect in the model. Our goal was to allow the data in an unsupervised fashion to be fit via the LCA method. Our findings are novel, specifically because of the analytical method.

### Limitations

The research design included a secondary data analysis that utilized a sample size of approximately 283 individuals with complete information on three questionnaires. As such, the sample size prevents (or limits) generalizability of the results as the secondary data analysis was not purposefully prospectively powered. Limited data points prevented in-depth statistical analysis. Additional care should be used in the interpretation of the findings as model fit analysis does not imply causation.

## Conclusions

Weight changes affect both GI and cardiovascular symptoms for PWHIV. As such, nutritional interventions may be beneficial for managing weight and reducing adverse effects for PWHIV [33]. Moreover, although the use of HAART medication is beneficial for treating and managing HIV infection, the long-term use of HAART medications may be problematic for healthy weight maintenance. Therefore, monitoring of weight status is important for PWHIV for reducing chronic disease conditions such as cardiovascular disease, hypertension, and diabetes; comorbid conditions that adversely affect health-related quality of life. Moreover, there is an unmet need for healthcare providers to recognize the symptomatic and comorbid factors that affect health outcomes in persons of diverse racial/ethnic backgrounds. Precision health initiatives that “take into account individual differences in people’s genes, environments and lifestyles,” and with the goal of “revolutionizing how we improve and treat disease,”[34] hold promise for healthcare treatments for PWHIV; such advances may indeed continue to prolong years of healthy living across the life span.

## Data Availability

The datasets used and/or analyzed during the current study are available from the corresponding author on reasonable request.

## Abbreviations

AIDS: Acquired immunodeficiency syndrome
BMI: Body mass index
CV: Cardiovascular
CD4: Cluster of differentiation 4
GI: Gastrointestinal
HAART: Highly active antiretroviral treatments
HIV: Human immunodeficiency virus
IBM SPSS: International Business Machines Corporation Statistical Package for the Social Sciences
JMP: John’s Macintosh Project
LCA: Latent class analysis
PWHIV: Persons with HIV
SAS: Statistical Analysis System
T-cell: Thymus cell
